# Mixture interactions at mammalian olfactory receptors are dependent on the cellular environment

**DOI:** 10.1038/s41598-021-88601-0

**Published:** 2021-04-29

**Authors:** Elizabeth A. Corey, Sergei Zolotukhin, Barry W. Ache, Kirill Ukhanov

**Affiliations:** 1 Whitney Laboratory, University of Florida, Gainesville, FL USA; 2 Department of Pediatrics, University of Florida, Gainesville, FL USA; 3 Department of Biology and Neuroscience, University of Florida, Gainesville, FL USA; 4 Department of Pharmacology and Therapeutics, University of Florida, Gainesville, FL USA; 5 Center for Smell and Taste, University of Florida, Gainesville, FL USA; 6 McKnight Brain Institute, University of Florida, Gainesville, FL USA

**Keywords:** Olfactory receptors, Cellular neuroscience, Sensory processing

## Abstract

Functional characterization of mammalian olfactory receptors (ORs) remains a major challenge to ultimately understanding the olfactory code. Here, we compare the responses of the mouse Olfr73 ectopically expressed in olfactory sensory neurons using AAV gene delivery in vivo and expressed in vitro in cell culture. The response dynamics and concentration-dependence of agonists for the ectopically expressed Olfr73 were similar to those reported for the endogenous Olfr73, however the antagonism previously reported between its cognate agonist and several antagonists was not replicated in vivo. Expressing the OR in vitro reproduced the antagonism reported for short odor pulses, but not for prolonged odor exposure. Our findings suggest that both the cellular environment and the stimulus dynamics shape the functionality of Olfr73 and argue that characterizing ORs in ‘native’ conditions, rather than in vitro, provides a more relevant understanding of ligand-OR interactions.

## Introduction

Mammalian olfactory receptors (ORs) form the largest gene family of all G-protein coupled receptors (GPCRs), with approximately 1100 members in rodents and 400 in humans^1^. Such a large number of receptors raises the challenge to understand the logic of olfactory coding. This fundamental question has been the renewed focus of a number of recent studies that have added to our understanding by showing that ORs encode information through broad-based excitation and inhibition^2–4^. This knowledge in turn adds weight to the importance of identifying the full response spectrum, i.e., the molecular receptive range (MRR), of individual ORs, including both the excitatory and inhibitory ligands.

ORs have proven difficult to deorphanize by expressing them in non-olfactory cells, in large part due to their poor surface expression resulting from the lack of several chaperon proteins, including small GTPases RTP1s, Ric8B and REEP1, which control OR trafficking from the trans-Golgi network to the plasma membrane^5^. Moreover, ORs are quite diverse and lack an identifiable consensus motif for membrane and olfactory cilia trafficking^6^. Some progress has been made by fusing a short lead sequence of the first 20 amino acids from bovine rhodopsin (Rho-tag) in-frame to the N-terminus of an OR to increase surface expression in cultured cells^5,7^. However, this approach has not proven successful for all ORs and other tags have been evaluated^8^.

The use of in vitro heterologous expression assays has led to the discovery and characterization of antagonistic interactions between structurally similar ligands for several mouse ORs, such as Olfr73, Olfr544, Olfr586 as well as one human OR17-4^9–11^. These assays have been developed to functionally characterize ORs utilizing coupling to the non-native promiscuous Gα15 protein leading to phospholipase-C activation or to the olfactory G_olf_ protein resulting in production of cAMP^7^. The G_olf_-dependent assay was further developed to allow high-throughput analysis utilizing the cAMP-dependent upregulation of a Cre response element fused to SEAP phosphatase or luciferase as a readout^12,13^. Remarkably, the high-throughput assay was successfully used to screen 176 mouse ORs to identify inverse agonists acting as putative inhibitory ligands^3^. It remains to be seen, however, if this assay would be as effective in identifying the complete MRR for any OR including both agonists and antagonists. Importantly, the findings of all in vitro studies should be verified in native OSNs^14^.

These constraints imposed by in vitro expression system have been partially alleviated by characterizing ORs in olfactory sensory neurons (OSNs) in vivo, e.g., using transgenic animals expressing a fluorescent protein or a reporter under the promoter of a given OR^14,15^. Several ORs of different classes have been successfully characterized using this approach^15–17^. However, given the sheer number of mammalian ORs, developing a transgenic line for each is not a practical approach. A less time-consuming approach has been to ectopically express ORs through virally assisted gene delivery to native OSNs. The rat receptor OR-I7 (Olr226, MOR103-15) was the first to be successfully characterized by this method^18^, leading in the follow-up study to the discovery of antagonistic ligands^19^. Subsequently, other recombinant viral vectors, including retrovirus and *Herpes simplex*, have been used to ectopically functionally express ORs and vomeronasal receptors in their respective sensory neurons^20–22^.

In this study, using the well-characterized mouse OR, Olfr73^10,13,17,23–25^ and recombinant adeno-associated virus (rAAV) vector-mediated gene delivery, we show markedly different responses when using two contrasting assay platforms, in vitro and in *vivo*. When ectopically expressed in vivo, Olfr73 mediated robust response to eugenol and other previously published agonists. However, a putative antagonist methylisoeugenol (MIEG) acted as weak agonist without antagonizing eugenol or vanillin. These findings mirrored those for mouse OSNs endogenously expressing Olfr73^26^ and opposing early findings^10,17^. Moreover, the same OR, heterologously expressed in vitro and challenged with prolonged application of two odorants, produced, surprisingly, an additive agonism to the otherwise antagonist odorant and not the predicted antagonism or lack of a response as reported previously^10,17^. Our findings argue that the functional output driven by Olfr73, and likely other ORs, is dependent on the innate cellular environment and stimulus dynamics, underscoring the need to characterize ORs in ‘native’ conditions in order to provide the most complete understanding of ligand-OR interactions.

## Results

### rAAV2/5 as a vector for gene delivery to mammalian OSNs

**Figure 1:**
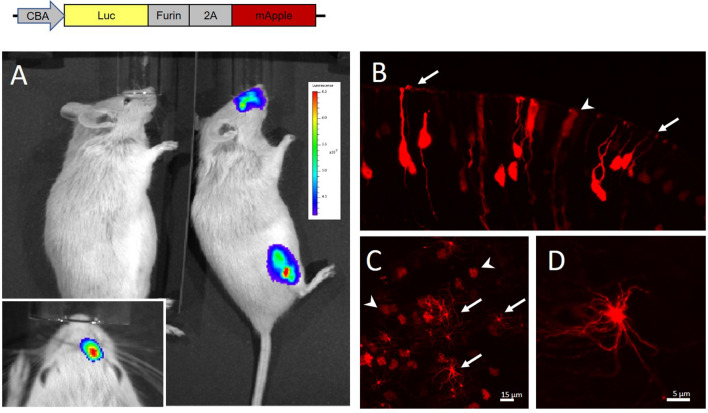
rAAV2/5 assisted gene delivery of Luciferase-furin2A-mApple induces persistent expression of the reporter proteins. (**A**) A single 20 µL injection of the rAAV2/5 vector induced ectopic expression of luciferase in the nasal cavity and tibial muscle. (Insert) Unilateral intranasal infusion of the vector confirmed restricted expression of the reporter. Control injection of sterile saline was used as a negative control (left animal). Pseudo-color scale shows intensity of luciferase bioluminescence from 3 to 6.5*10^7^ photons per second. (**B**) Coronal section of postfixed OE from the same animal used for in vivo imaging in (**A**). rAAV2/5 induced expression of mApple in OSNs (arrows) and in sustentacular cells (arrowheads). (**C**) *En face* images of the freshly dissected OE showing groups of rAAV2/5 induced OSNs (arrows) and non-sensory sustentacular cells (arrowheads). (**D**) Magnified image of the dendritic knob decorated with numerous cilia.

Given limited prior knowledge of the tropism of different AAV serotypes in the mammalian nasal epithelium, we tested an rAAV5-based vector not reported previously to specifically transduce OSNs but known to display a broad tissue tropism^27^. A bicistronic cassette encoding firefly Luciferase and mApple fluorescent protein linked by the furin-cleavage signal and ribosome skipping peptide 2A from a foot-and-mouth virus (Fig. 1A, top), was used to produce the rAAV5 vector^28^. A single dose of rAAV5 (10–20 µl, titer ca. 10^11^ vg/ml) was administered intranasally to anesthetized rats and mice. Robust Luciferase activity, restricted to the mouse nasal cavity only, was detected 7 days later (Fig. 1A). The same animal used for in vivo Luciferase imaging was subsequently transcardially perfused and analyzed for the mApple expression in the olfactory epithelium (OE) (Fig. 1B). rAAV5 successfully induced expression in multiple OSNs along with non-sensory sustentacular cells (Fig. 1B). Similarly, *en face* imaging of the acutely isolated OE revealed a mixed population of transduced OSNs and sustentacular cells (Fig. 1C). At higher magnification multiple cilia emanating from the dendritic knob of the OSN could be clearly resolved (Fig. 1D).

### rAAV5-mediated transduction of the olfactory epithelium and ectopic expression of the OR in rodent OSNs

**Figure 2:**
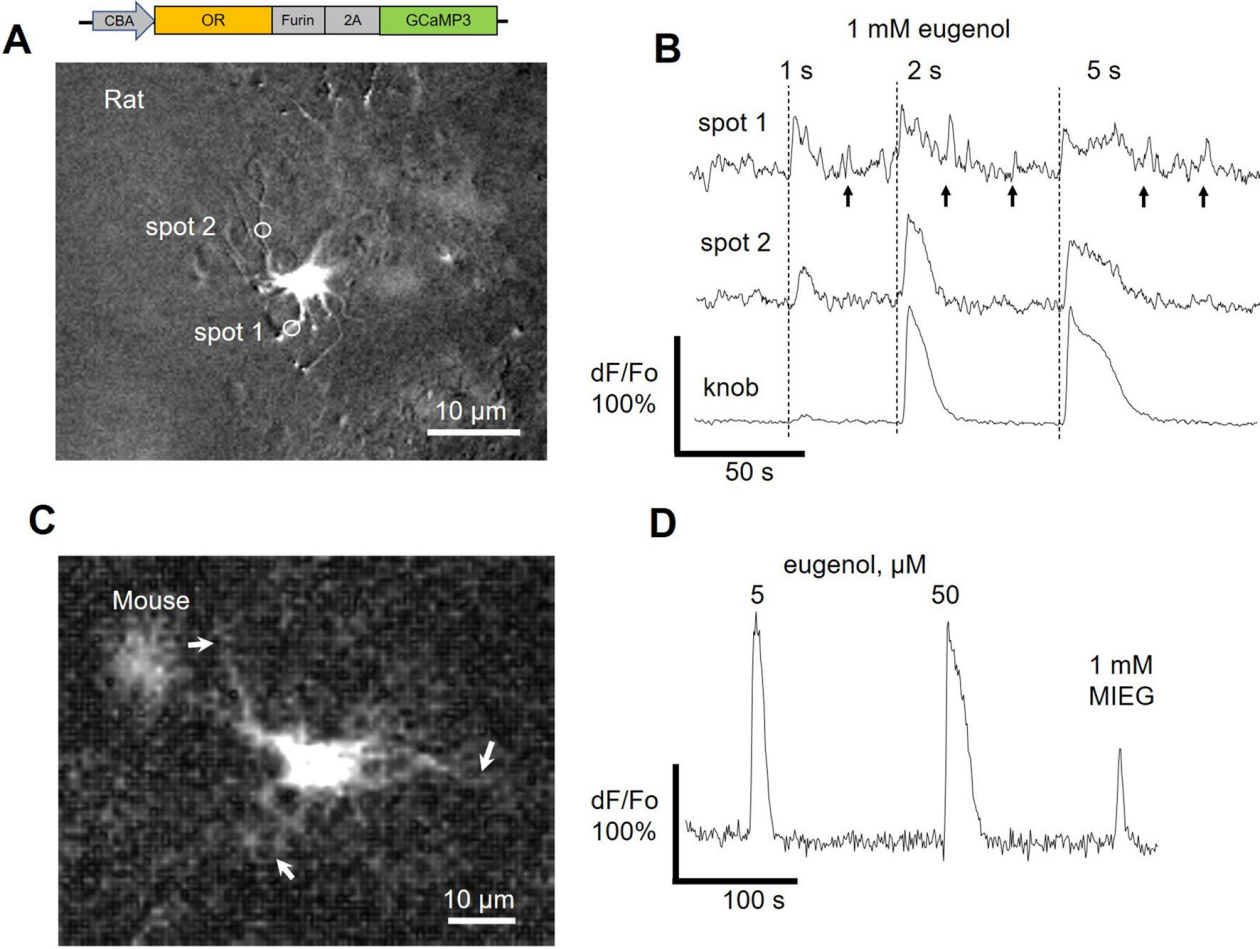
Robust eugenol-evoked activation of mammalian OSNs ectopically expressing Olfr73. Neuronal activity was measured with GCaMP3 expressed from the same rAAV2/5 vector along with the OR. (**A**,**B**) A single knob of the rat Olfr73-OSN which was stimulated with a solution containing 1 mM eugenol with pulses of increasing duration. A short 1-s long pulse activated nearly threshold response in the knob (bottom trace). However, in two representative cilia the same stimulus elicited much stronger relative increase of GCaMP3 fluorescence (spot 1 and 2). Note, activity persisted as reflected by a transient intraciliary Ca^2+^ fluctuations (arrows, upper trace). (**C**,**D**) Mouse Olfr73-OSN was strongly activated by a low concentration of eugenol (5 µM, 5-s pulse). Application of methylisoeugenol (MIEG, 1 mM, 5-s pulse) also elicited a response. Cilia attached to the knob can be clearly resolved (arrows).

Following initial characterization of transduction of the OE by rAAV5, we subcloned full-length Olfr73, a Class II OR (a.k.a. MOR174-1, mOR-EG)^1^ and a genetically-encoded calcium indicator GCaMP3 in to the same vector by substituting the respective ORFs (Fig. 2A, top). We hypothesized that OSNs should have all necessary endogenous cellular mechanisms for the correct expression and trafficking of the OR protein to cilia. Hence, no additional modification of the OR gene via fusing the N-terminus tag to improve plasma membrane localization was made, thus ensuring that expression in OSNs is identical to its natural counterparts.

While we could not exclude that a few residues of the furin signal remain after cleavage, this did not appear to impose a serious problem for measuring antagonism between eugenol and MIEG since the bicistronic cDNA was functional in vitro. Functional expression of the OR and GCaMP3 was validated in HEK293T cells by co-transfecting the pTR plasmid encoding the bicistronic cDNA used to produce rAAV5 with the promiscuous G-protein Gα15, conferring coupling to the downstream calcium release signaling cascade. A 5 s application of eugenol (100 µM) evoked robust elevation of intracellular calcium (Supplemental Fig. S5A). Our result confirmed earlier reports of functional expression of untagged full-length Olfr73 in HEK293 cells^29^ and demonstrated the functionality of the bicistronic expression cassette.

**Figure 3:**
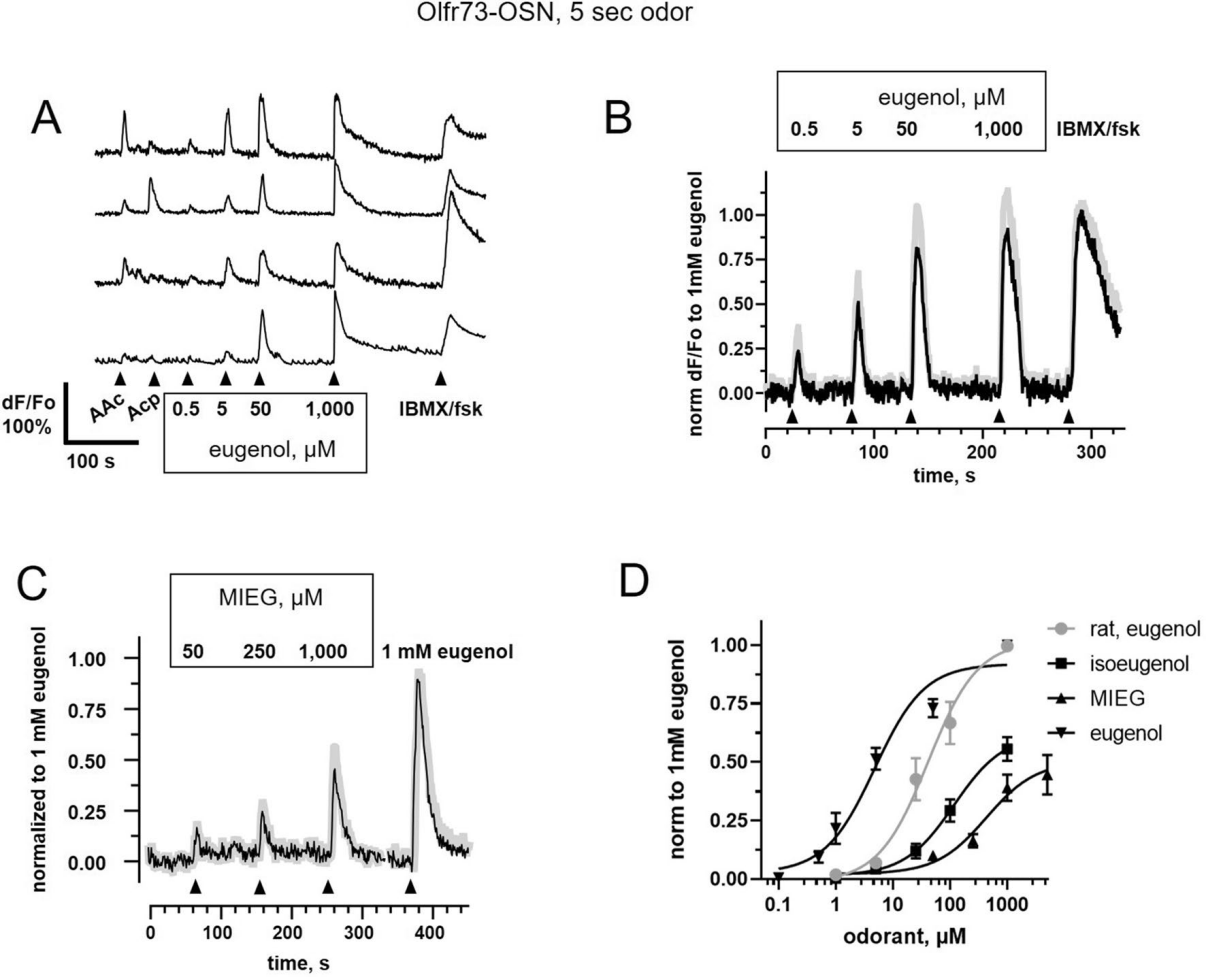
Characterization of the response to eugenol measured in Olfr73-OSNs in mouse OE in situ. (**A**) Typical profile of the response elicited by application for 5 s of two distinct from eugenol odorants, amyl acetate (AAc) and acetophenone (Acp). Each of the selected OSNs reacted differently to AAc and Acp (each at 100 µM). However, increasing concentration of eugenol evoked a dose-dependent response in all 11 cells in this group. A solution containing IBMX/forskolin (IBMX/fsk, 100/10 µM) was used as a positive control activating functional canonical OSNs. (**B**,**C**) The response measured in a group of different 6 cells sequentially stimulated with increasing concentration of eugenol, followed by methylisoeugenol (MIEG, concentration of ligand in µM is indicated above each trace). After applying MIEG, a pulse of eugenol (1 mM Eug) was given as a positive control. Averaged traces are shown with SEM superimposed as a gray shadow. (**D**) Summary of the dose–response profiles of the mouse and rat Olfr73-OSNs. Data points were fitted to the Hill equation yielding the following EC_50_ values (41 ± 12 µM eugenol, rat, gray circles, n = 13; 4.8 ± 1.1 µM eugenol, inversed triangles, n = 24; 117 ± 45 µM, isoeugenol, squares, n = 24; 430 ± 230 µM, MIEG, triangles, n = 15). The data were collected from 6 mice and 2 rats and shown as mean ± SEM.

Next, single intranasal doses of rAAV5 were used to induce expression of the bicistronic construct in mouse and rat OEs, as evidenced by the fluorescence of GCaMP3 in many cells at a level sufficiently strong to visualize dendritic knobs with their attached cilia (Fig. 2). This allowed visualization of the functional activity within the transduction compartment in semi-intact tissue with high resolution (Supplemental movie S1, S2). In response to a brief 5-s pulse of eugenol (1 mM), we observed an odor-evoked global cilia-to-knob response, as well as residual activity in the cilia post-stimulation (Fig. 2B, arrows). These results clearly suggest that both Olfr73 and GCaMP3 were localized to the transduction compartment. Transduced mouse OSNs responded to lower concentrations of eugenol (5 µM) and showed left-shifted concentration–response functions compared to rat OSNs (Figs. 2D; 3D). Methylisoeugenol (MIEG, 1 mM), a previously characterized antagonist of eugenol on Olfr73^9^, also evoked a measurable response. The latter finding confirmed a similar outcome previously reported in mouse OSNs endogenously expressing Olfr73^26^ and set the foundation of the current study.

To confirm the general utility of the rAAV5 delivery and expression system we subcloned another mouse OR, a Class I Olfr599 (MOR23-1), using the same bicistronic cassette (Fig. 2A, top). First, we verified that Olfr599 was functionally expressed in our expression system either as a rho-tagged OR or as the untagged OR. Octanoic acid, a ligand of the OR^12^ consistently activated a response in a concentration-dependent manner with an EC_50_ = 70 µM (n = 21) (Supplemental Fig. S1A,B). Importantly, transfection of untagged Olfr599 using the bicistronic pTR-Olfr599-furin2A-GCaMP3 plasmid also resulted in functional expression of the OR along with the calcium reporter (Supplemental Fig. S1C). Similarly, intranasal infusion of rAAV2/5-Olfr599-furin2A-GCaMP3 induced expression as evident by GCaMP3 fluorescence in the knobs of mouse OSNs (Supplemental Fig. S1D). Octanoic acid and octanal (each at 100 µM), as well as a mix of IBMX and forskolin (100/10 µM), a chemical activator of most OSNs, all evoked reliable neuronal responses (Supplemental Fig. S1E).

Finally, we asked whether rAAV-mediated potential over-expression of ectopic ORs could have induced an elevated basal activity known to be dependent on the type of the OR^30^. As a proxy for basal neuronal activity, we counted Olfr73-GCaMP3-OSNs (Olfr73-OSNs) showing aberrant calcium bursts as previously published^31^. In the entire pool of Olfr73-OSNs, we found only 6.44% (13 of 202 cells) spontaneously active Olfr73-OSNs, generating bursts at 0.08 ± 0.03 Hz (n = 13). These values were even lower than 12.9% of unidentified OSNs bursting at 0.16 ± 0.01 Hz reported in a recent study^31^. This confirms previously published data of low basal activity of native Olfr73-ires-tauGFP OSNs, as well as Olfr73 expressed in HEK293 cells^3,30^. Overall, our results validated the prior understanding that ectopic expression of a different OR in a mature OSN can be used as a reliable model to study ORs in the native cellular environment^18^.

### Ligand profile of OSNs ectopically expressing Olfr73

**Figure 4:**
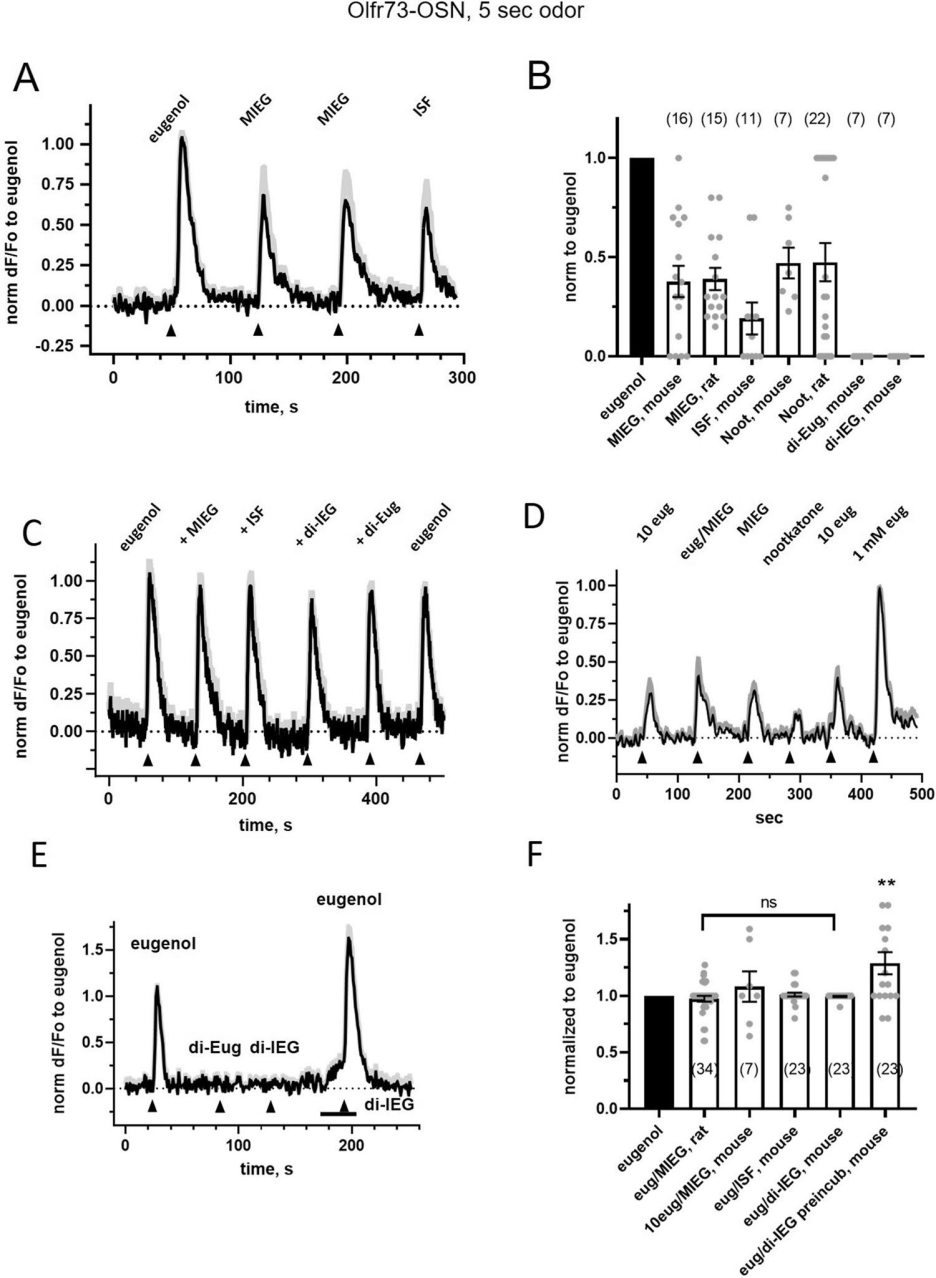
Putative antagonists of Olfr73 methylisoeugenol, isosafrole and dimerized isoeugenol did not diminish the response to eugenol in rodent Olfr73-OSNs. (**A**) Mouse OSNs activated by eugenol (100 µM), methylisoeugenol (MIEG, 1 mM) and isosafrole (ISF, 1 mM). (**B**) Response elicited by application for 5 s of each ligand was normalized to that of eugenol (100 µM). MIEG and nootkatone similarly activated mouse and rat OSNs. Dimerized eugenol (di-EG) and isoeugenol (di-IEG) applied at 500 µM did not elicit any response (see also **E**). Number of cells in each set is shown above each bar. (**C**) Binary mix of eugenol (100 µM) and a known putative antagonist (same concentration as in **B**) was applied to mouse OSNs. After the test, eugenol (100 µM) was applied again to account for any run-down. (**D**) MIEG at the same high concentration (1 mM) did not reduce the response when co-applied with a low 10 µM eugenol. A different putative high potency agonist nootkatone was tested at 1 mM (**E**) Dimer of eugenol (di-Eug) and isoeugenol (di-IEG) both at 1 mM did not evoke any response. Co-application of di-IEG was not able to antagonize the response elicited by eugenol (100 µM) even following preincubation for 30-s (bar below the trace). Individual traces were averaged in each respective group of 5–8 cells and shown with SEM superimposed as a gray shadow. (F) Response evoked by a different binary mix were normalized to that evoked by eugenol alone. Wilcoxon paired test, **p = 0.017, non-significant (ns). Number of cells used in each set is shown inside bars. At least three animals were used to collect data for each ligand pair. Data are shown as mean ± SEM.

First, we confirmed that viral ectopic expression of Olfr73 in the OE conferred its previously characterized MRR. Olfr73-OSNs were challenged with two ligands outside the known MRR of Olfr73, amyl acetate and acetophenone (both at 100 µM), followed by increasing concentrations of eugenol. Amyl acetate and acetophenone evoked variable responses in different Olfr73-OSNs, reflecting the expected expression of different endogenous ORs in these cells (Fig. 3A), while application of eugenol consistently evoked a response in all cells in a concentration-dependent manner (Fig. 3B). In each case, the response profile matched that expected for Olfr73 (Fig. 3B). The agonists eugenol and isoeugenol evoked responses in mouse Olfr73-OSNs in a concentration-dependent manner, yielding EC_50_ values of 4.8 ± 1.1 µM (n = 18) and 117 ± 45 µM (n = 24), respectively (Fig. 3B,C; Table 1). Rat Olfr73-OSNs showed a similar response profile but were nearly tenfold less sensitive to eugenol than mouse OSNs, yielding a right-shifted dose response curve with an EC_50_ of 41 ± 12 µM (n = 13) (Fig. 3D). MIEG acted as a partial agonist for Olfr73 expressed in native mouse OSNs, yielding an EC_50_ of 430 ± 130 µM (n = 15) (Fig. 3D). Isosafrole, a putative antagonist of Olfr73^23^, even applied at high concentration (1 mM), only activated Olfr73-OSNs at 25% (ISF, n = 11). We also tested nootkatone, a putative highly potent agonist of Olfr73 discovered in in vitro assay^13^ which turned out to be a weak agonist for Olfr73-OSNs eliciting a response at 50% (Noot, n = 7) of the response to eugenol (1 mM) (Fig. 4A,B,D). The response of rat and mouse Olfr73-OSNs to nootkatone was lower than that to eugenol (Fig. 4B), suggesting that in native OSNs nootkatone acts as a weak agonist.

**Table 1:** Agonism and antagonism of several ligands of mouse Olfr73 is dependent on the assay and stimulation.

Ligand	OSN Ca^2+^		HEK293 long odor Cre/SEAP Ca^2+^		HEK293 short odor Ca^2+^		
	Mouse	Rat	Gs/olf		Gs/olf	G15	Gq11
Eugenol	**4.8±1.1**	**41 ± 12**	**205 ± 27**	**Agonist**	**Agonist**	**Agonist**	**184 ± 62**
Isoeugenol	**117 ± 45**				Agonist*	Agonist*	**23 ± 8**
Vanillin					**Agonist**	**Agonist**	**130 ± 73**
No otkatone Nootkatone	**Agonist**	**Agonist**			Inactive*	Inactive*	
Methylisoeugenol	**430 ± 130**	**Agonist**	**Agonist**	**Agonist**	*Antagonist*	*Antagonist*	*Antagonist*
Isosafrole	**Agonist**				*Antagonist*	*Antagonist*	*Antagonist*
di-Isoeugenol	Inactive				*Antagonist*	*Antagonist*	*Antagonist*
di-Eugenol	Inactive				*Antagonist*	*Antagonist*	*Antagonist*

Changes of intracellular Ca^2+^ were used to measure activation of mammalian Olfr73-OSNs evoked by a short 5-s odorant pulse. Cre/SEAP reporter assay or cAMP-dependent influx of Ca^2+^ were used to measure activation of HEK293 cells expressing Olfr73 following prolonged 30-min and 10-min odorant application, respectively. Changes of intracellular Ca^2+^ were used to measure activation of HEK293 cells expressing Olfr73 coupled to a respective G-protein and following a short 5-s odorant pulse. EC_50_ values (µM ± SD) of several ligands are shown in the respective boxes. Ligands which showed agonism on Olfr73 are labeled in bold and those showed antagonism are labeled in italic. A strong agonist (eugenol, vanillin) was presented at 100 µM and a putative antagonist at 1 mM as a single compound or as a pair mixed at these concentrations. *For comparison purpose isoeugenol and nootkatone were listed as agonist and inactive odorant based on the previously published in vitro work^25^, performed under the same conditions as in the current study.

### Ectopically expressed Olfr73 does not mediate ligand-dependent antagonism in native OSNs

Interaction of ligands in binary mixtures may result in antagonism even though one or both of the components alone acts as a low-potency agonist^32,33^ so we sought to further characterize the interaction between eugenol and several potential antagonists. First, we established the response profile of the Olfr73-OSNs to repeated stimulation with a 5-s pulse of eugenol (100 µM) followed by the pulse of the putative antagonist (1 mM) (Fig. 4A). While MIEG evoked responses of similar amplitude relative to eugenol in both rat and mouse Olfr73-OSNs (Fig. 4B), we limited these experiments to mouse OSNs to exclude any issues related to the reduced sensitivity of rat Olfr73-OSNs (Fig. 3D). In addition to MIEG and isosafrole, we tested the chemically dimerized isoeugenol (di-IEG, 500 µM), identified as an antagonist of eugenol on Olfr73^23^. In contrast to MIEG and isosafrole, di-IEG, as well as a dimer of eugenol (di-Eug, 500 µM) failed to activate any response in mouse Olfr73-OSNs (Fig. 4B,E). Application of the putative antagonists in a binary mixture with eugenol also failed to show any inhibition or additivity relative to the control response evoked by eugenol alone (Fig. 4C,F). Furthermore, the response to eugenol at its EC_50_ concentration (10 µM) mixed with MIEG (1 mM) was not significantly changed from the control response to eugenol alone (Fig. 4D,F), suggesting that pre-incubation with a putative antagonist may impose stronger inhibition. Both dimers, di-Eug and di-IEG (500 µM), failed to elicit a response when applied alone for 5-s (Fig. 4B,E). However, pre-incubation for 30-s with di-IEG elicited a small increase of the GCaMP3 signal, with no attenuation of the response to the binary mix with eugenol (Fig. 4E). Co-application of di-IEG resulted in an additive increase when mixed with eugenol (Fig. 4F), suggesting that the dimers of eugenol and isoeugenol did not antagonize the response to eugenol. Overall, we failed to identify any antagonistic or inhibitory interaction of eugenol and its putative antagonists in native mouse OSNs ectopically expressing untagged Olfr73.

### In vitro-expressed Olfr73 mediates antagonism between ligands independently of the downstream signaling pathway

**Figure 5:**
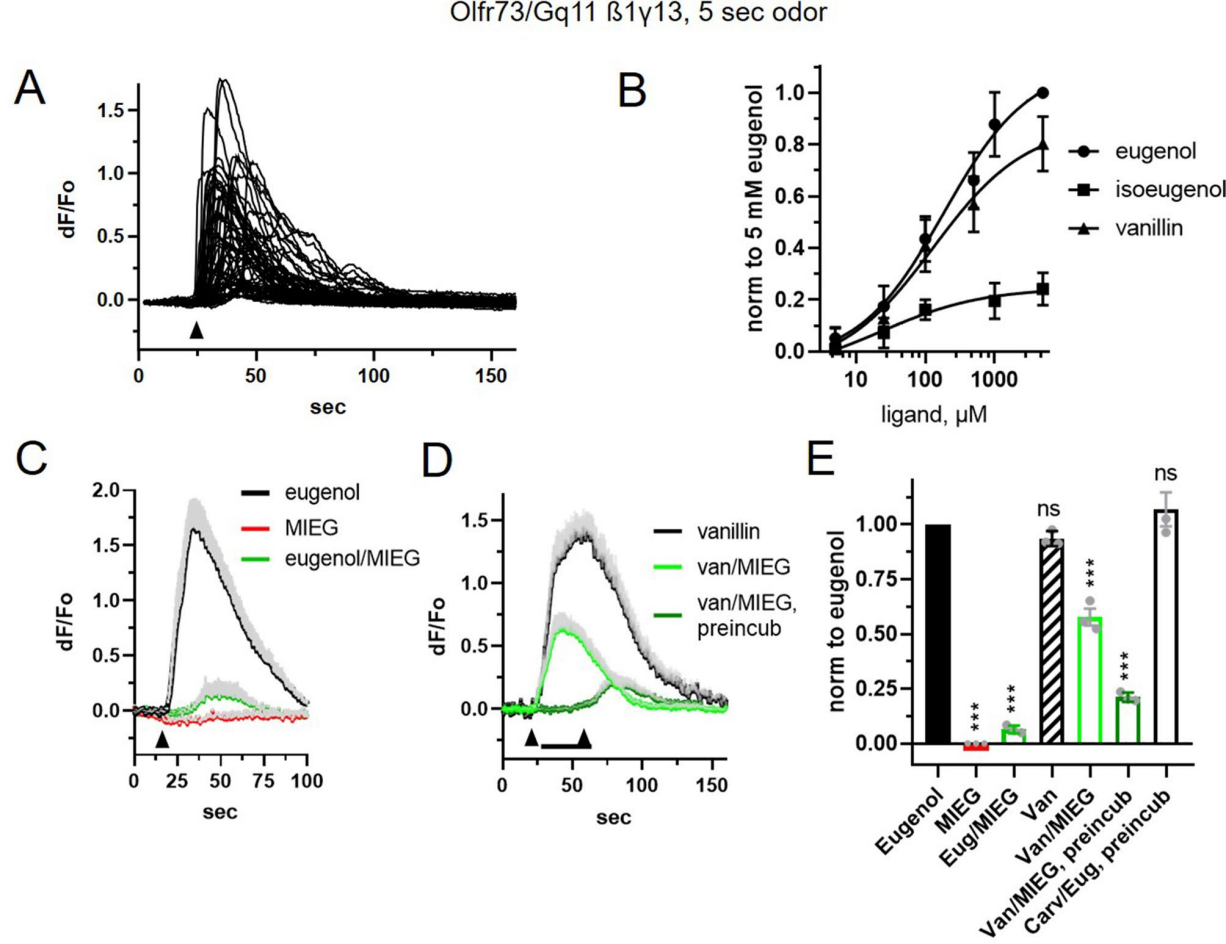
Co-expression of Olfr73 with a native heteromeric G-protein Gq11/b1/g13 does not change the antagonism in the binary mix of agonists and putative antagonists. (**A**) Co-expression of Olfr73 with a complete heterotrimeric G-protein Gq11 couples the OR to the endogenous PLC-dependent calcium release pathway and evokes transient increase of cytoplasmic Ca^2+^ following a 5-s application of eugenol (100 µM) indicated by arrow below the traces. The eugenol-evoked change of intracellular Ca^2+^ was measured individually in 25 cells. (**B**) Concentration-dependence of the response elicited by three Olfr73 agonists. The response represents changes of intracellular Ca^2+^ normalized to that evoked by 5 mM eugenol. Data points were fitted to the sigmoidal curve yielding the following EC_50_ values (184 ± 62 µM, eugenol, circles; 23 ± 8 µM, isoeugenol, squares; 130 ± 73 µM, vanillin, triangles). Data were collected from 25–30 cells in 3 independent experiments. (**C**) Binary mix of eugenol (100 µM) and MIEG (1 mM) as well as ligands alone were applied at indicated time (arrowhead). (**D**) Another potent agonist, vanillin (100 µM) was antagonized by methylisoeugenol (MIEG, 1 mM) but required pre-incubation for 30-s to achieve near complete inhibition. Traces represent mean ± SEM measured in 20–30 cells. (**E**) Responses in all groups were normalized to the control response evoked by eugenol alone. Carvone (1 mM, Carv) was used as a negative control acting as a non-agonist and imposing no antagonism in the mix with eugenol. Data represent three independent experiments. Wilcoxon paired t-test was applied, *p = 0.017; **p = 0.0014; ***p = 0.0001. Data are shown as mean ± SEM.

Since previous research suggested that antagonism in binary mixtures of ligands may depend on the signaling pathway downstream of the OR in question^9^, we explored the effect of substituting different G proteins in our in vitro assay. First, we confirmed that mammalian ORs can robustly couple to endogenous stimulatory signaling pathways, mediated not only by Gs/olf but also by Gq11, found in ciliary proteome in mammalian OSNs^34^. Eugenol (100 µM) consistently activated calcium release in HEK293 cells co-expressing Olfr73 with the complete heterotrimeric G-protein, Gαq11/ß1/γ13 (Fig. 5A). Using this expression system, we then measured the concentration-dependence of the response to eugenol, vanillin and isoeugenol (Fig. 5B). Co-expression of another mouse OR, Olfr599 with the heterotrimeric G-protein, Gαq11/ß1/γ13, also conferred robust activation by its cognate ligand, octanoic acid (100 µM), as was the case for Gs/olf and Gα15-dependent signaling (Supplemental Fig. S2). Thus, mammalian ORs have the capacity of coupling to the heterotrimeric Gq11/ß1/γ13 G-protein naturally expressed in olfactory cilia.

We then characterized the interaction between cognate agonists and putative antagonists of rho-tagged Olfr73 co-expressed with either Gαq11/ß1/γ13, Gs/olf, or the promiscuous G protein, Gα15. We not only validated the earlier finding that Olfr73 transiently expressed in HEK293 cells conferred the antagonism imposed by MIEG on the response to eugenol but also found that its antagonism could be imposed on another strong agonist, vanillin (Fig. 5; Supplemental Fig. S3, S4). Interestingly, the response to vanillin (100 µM) was only partially inhibited by co-application of MIEG (1 mM), however it was almost completely blocked following 30-s pre-incubation with MIEG (Fig. 5D,E; Supplemental Fig. S3, S4). Carvone (1 mM), distantly structurally related to eugenol but a non-agonist for Olfr73^33^, failed to antagonize the response to eugenol even following pre-incubation for 30-s (Fig. 5E; Supplemental Fig. S3, S4). These findings argue that antagonistic interactions between cognate agonists and putative antagonists can occur independently of the signaling pathway which is downstream of ORs in vitro.

### Interaction of binary mixtures with the in vitro-expressed Olfr73 is dependent on the stimulus paradigm

**Figure 6:**
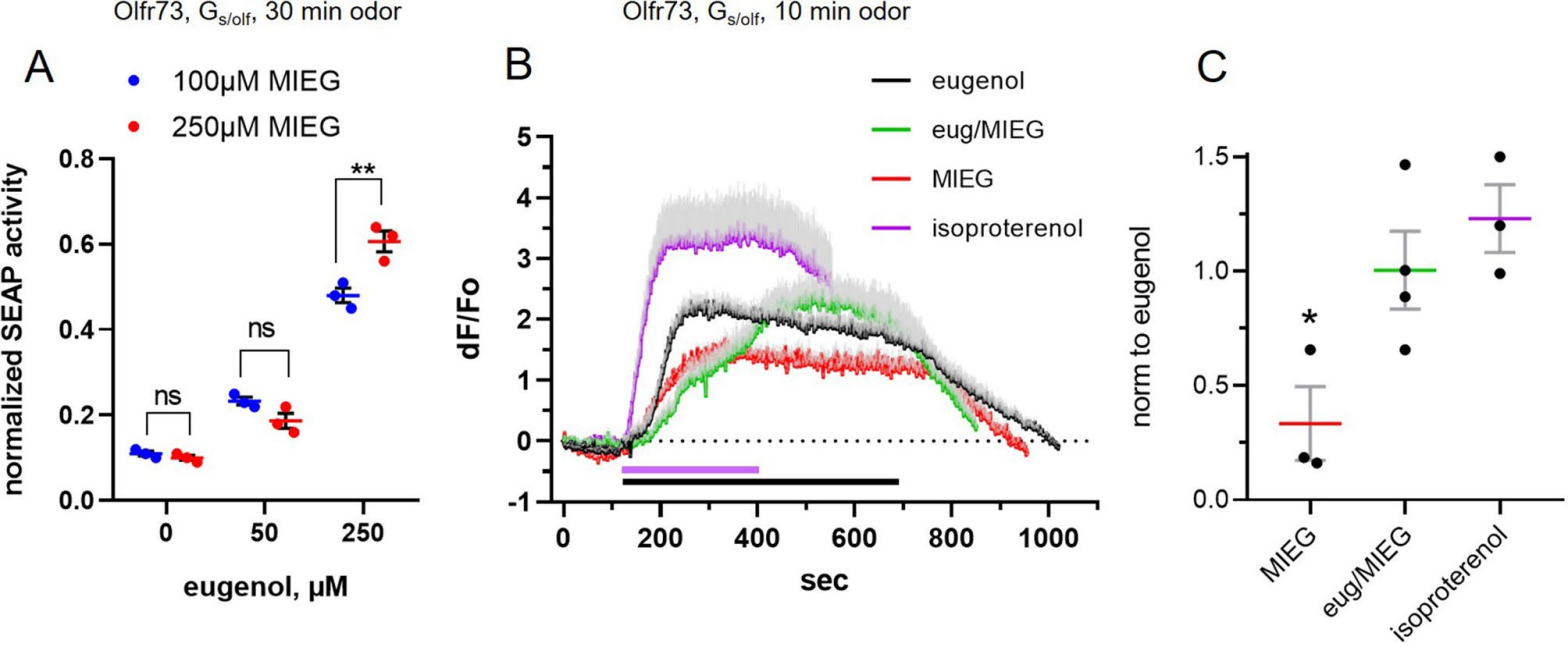
Prolonged exposure to Olfr73 ligands activates sustained cAMP build-up and reveals absence of antagonism in a binary mix of eugenol and methylisoeugenol. (**A**) HEK293 cells co-expressing untagged Olfr73 generated cAMP following a 30-min incubation with methylisoeugenol (MIEG) alone or its mixture with eugenol. cAMP was measured indirectly by the activity of Cre-SEAP (multiple t-test, p = 0.288 (0 µM eugenol); p = 0.077 (50 µM eugenol); **p = 0.013 (250 µM eugenol)). (**B**) Time-resolved cAMP-dependent activity directly measured by GCaMP3 as a Ca^2+^ influx in HEK293 cells co-expressing untagged Olfr73 and CNGCmut channel, a cAMP sensor. Eugenol (100 µM), MIEG (1 mM) and their binary mix were applied for 10 min at the time indicated (arrowhead). Isoproterenol (10 µM) was applied for 5 min at the end of the experiment to confirm the functionality of cells and that the response to ligands was not saturated. Traces represent average (solid line) and SEM (gray shadow) measured in the same group of 26 cells. (**C**) Ligand induced cAMP-dependent activity was measured in at least three independent experiments at the plateau of the response and normalized to that evoked by eugenol alone. Wilcoxon paired t-test, *p = 0.018 (MIEG, n = 3); p = 0.875 (eug/MIEG, n = 4). Data are shown as mean ± SEM.

We used the Cre-SEAP reporter assay, similar to the more widely used Cre-Luciferase reporter assay^5,13^, to measure cAMP generated by eugenol applied for a prolonged time to HEK293 cells co-expressing untagged Olfr73 and Gs/olf (Supplemental Fig. S6). MIEG alone also generated cAMP underscoring our finding that in this assay a putative antagonist acts as an agonist (Fig. 6A, at 0 µM eugenol). Surprisingly, we observed no antagonistic interaction between several concentrations of eugenol and MIEG, showing instead an increase in net activity evoked by incubation with increasing concentration of the binary mix (Fig. 6A). In order to address whether the stimulus protocol shaped the result, we co-expressed the rAAV2/5-targeting plasmid pTR-Olfr73-furin2A-GCaMP3 along with Gα15 in HEK293T cells. In this context, eugenol (100 µM) applied for 5 s elicited a robust calcium signal that was significantly diminished after co-application of MIEG (1 mM). MIEG alone did not elicit any response (Supplemental Fig. S5).

Finally, to address the issue of prolonged stimulus duration, we directly measured kinetics of cAMP-dependent activity in cells co-expressing untagged Olfr73 along with Gs/olf and CNGCmut channel as a cAMP sensor. To ensure that prolonged incubation with ligands did not saturate the cAMP assay readout, we used isoproterenol (10 µM) as a saturating stimulation. Isoproterenol applied for 5-min activated endogenous adrenergic receptors coupled to Gs/cAMP signaling and generated a robust sustained response exceeding any odorant-evoked response (Fig. 6B). Eugenol (100 µM) applied for 10-min generated a sustained cAMP-dependent calcium influx (Fig. 6B; Supplemental movie S3). A 10-min application of MIEG (1 mM) evoked a more slowly developing cAMP-dependent calcium influx (Fig. 6B). Averaged across several independent measurements the response evoked by MIEG alone was significantly smaller than eugenol (Wilcoxon test, *p = 0.018, n = 3). Importantly, however, a response evoked by application for 10 min of a mixture of eugenol (100 µM) and MIEG (1 mM) showed no significant difference from the response evoked by eugenol alone (Wilcoxon test, p > 0.9999, n = 4) (Fig. 6C; Supplemental movie S4). Thus, two independent methods of detecting Olfr73-activated cAMP-dependent activity confirmed that prolonged stimulation of the OR expressed in vitro results in agonism by the same ligand that with brief stimulation is a potent antagonist.

## Discussion

In the current study, we find that ligands previously characterized as antagonists for Olfr73^10^ behave as partial agonists in vivo in native OSNs ectopically expressing the receptor. Additional in vitro antagonist, dimerized isoeugenol^23^, behaved in our in vivo study as a neutral ligand, neither agonist nor antagonist. While MIEG was initially characterized as an antagonist, a subsequent study based on imaging of presynaptic odor-evoked activity in the olfactory bulb of the synapto-pHluorin-GFP mouse failed to detect any antagonistic interaction in a binary mixture of eugenol and MIEG^36^. Furthermore, a more recent study using electrophysiological patch-clamp recording from intact OSNs endogenously expressing optically tagged Olfr73, found that MIEG behaved as a weak agonist and failed to antagonize the response to eugenol and another agonist, vanillin, in binary mixtures^26^. It is possible that these opposing results originate not only from using a different methodology but also the age of animals. In the initial in vivo studies, dissociated mouse OSNs were isolated from adult 4–6 week old mice^10,17^, whereas the later study used more intact neurons in acute coronal epithelial slices from young P2-5 mice^26^ at a time when the cellular development and OR tuning are not yet established^37^. However, our current study argues that Olfr73 ectopically expressed in native OSNs of adult animals fully recapitulates the findings of^26^. Thus, it appears that in the innate OSN cellular environment, MIEG behaves as a true agonist for Olfr73.

Since we validated the earlier finding that MIEG and structurally similar ligands (isosafrole and dimerized isoeugenol) antagonize the response to eugenol when Olfr73 is expressed in vitro in HEK293 cells^10,23^, we would suggest that the differences that we found when comparing the results of in vivo vs in vitro expression were not procedural, but instead the result of OR expression in the absence of its native accessory signaling elements. This conclusion is supported by the finding that antagonism between eugenol and MIEG was also observed in a non-mammalian in vitro expression system, *Xenopus* oocytes, in which Olfr73 was co-expressed with G_olf_ and a cystic fibrosis transmembrane conductance regulator as a cAMP sensor^24^, and it suggests that ORs can mediate similar in vitro responses to ligands independent of the cell type used. This possibility receives further support from our finding that the antagonism measured in vitro was independent of the signaling pathway mediating the agonist-induced response. However, it should be stressed that the structure-response activity of the same OR may differ depending not only on the cellular environment (native vs heterologous) but also on the G-protein subtype and other accessory factors coupled to the OR, as has been reported previously in multiple studies of mouse Olfr73^13,25,38,39^. Indeed, in our study we found that nootkatone, a ligand identified as a highly potent agonist of Olfr73 in one in vitro assay^13^, acted as a weak agonist in mouse and rat Olfr73-OSNs and yet completely failed to elicit a signal in other in vitro assays^25^. Further evidence supporting the inconsistent ligand ranking for mammalian ORs measured in vitro and in vivo has been reported in a study performed on mouse OSNs endogenously expressing optically tagged ORs^40^.

The idea that the innate cellular environment critically determines the response properties, and thus the ligand ranking, of an OR aligns with a number of studies of other GPCRs^41^, including the finding that pilocarpine, a well-known therapeutic drug and agonist of muscarinic receptors, can act as either an agonist or antagonist on the M3 receptor depending on the cell type, the expression level, and the signaling pathway coupled to the receptor^42^. Moreover, it is now well documented that a ligand acting on a single GPCR species can impose multiple intrinsic efficacies. In other words, a ligand can be an agonist or antagonist when acting on the same receptor expressed in different cellular environments either in vitro or in vivo^43^. An important element that shapes signaling bias is the stochiometric ratio of the different signaling molecules that are able to bind to activated receptor conformation^44^, which one can assume is likely to vary significantly among different cell types and tissues. Following this logic, the OR-ligand interaction could be governed by a unique complement of endogenous chaperons or guest molecules orchestrating the outcome of the receptor activation in olfactory cilia as proposed earlier for GPCRs in general^45,46^. This is especially relevant given that the olfactory cilia constitute a specialized cellular compartment with a highly specialized tubular architecture and distinct plasma membrane lipid composition^47^. Even at the level of native OSNs, the ligand ranking for the same OR may not be the same in different animal species as our data clearly shows a tenfold difference in sensitivity between rat and mouse OSNs expressing Olfr73. It would be instructive to ask if a non-mouse OR, for example rOR-I7 (Olr226) expressed in mouse OSNs would confer its native tuning or would show a species-dependent shift in its sensitivity.

Our study does not address how the ligand-OR interaction leads to the ligand ranking of individual odorants or to mediate the agonism/ antagonism identified in some mixtures, but it likely results from one or more of the mechanisms identified for other GPCRs^48^. One possibility is that some ligands sharing common molecular features may bind to the OR with varying strengths or occupation times, resulting in different levels of signal strength. In this case, such ligands in binary mixtures may antagonize one another through direct competition for the binding site. Another possibility is antagonist binding to an allosteric site on the OR resulting in suppression of agonist signaling. Since ORs belong to class A GPCRs and allosteric ligands have been reported for class A and C GPCRs^49,50^ it is plausible that such interactions are likely to occur on ORs^32,51^. Some GPCRs have been found to activate complex signaling networks and, importantly, adopt multiple conformation states upon agonist binding^52^. Such a situation may be applicable to ORs since non-antagonistic ligands for Olfr73 as well as other ORs expressed in vitro act as biased agonists preferentially activating the downstream signaling pathway to which they were coupled^9,25^. In some cases, indeed, ligands may act as antagonists on one signaling pathway while simultaneously being agonists for another^53,54^. Importantly, biased agonism in olfactory signaling has been partially documented in vivo^55^. Clearly these potential mechanisms will be important to explore going forward given evidence that non-competitive as well as competitive interactions can mediate odorant mixture interactions^3,32,56^.

Our finding that the antagonism observed in vitro under short term stimulation was relieved by prolonged stimulation is consistent with prior evidence that Olfr73 manifested a dramatically altered structure–activity relationship measured in a cAMP-dependent Cre reporter system critically dependent on the duration of stimulation^12^. It is also consistent with the more general concept that long-term exposure of GPCRs to selective ligands may change occupancy of the receptor and result in different output^57^, which we would suggest can explain the observed time dependency of stimulation. This finding points to the need to characterize the pharmacological profiles of ORs expressed in vivo in native OSNs and to mimic the natural dynamics of stimulation.

As our field begins to define the MRRs of a wide range of mammalian ORs using high throughput in vitro assays^12,58^, it is important that putative agonists and antagonists for a given OR are validated in vivo in the native animal species. Going forward, our findings argue that a combination of ectopic viral expression of defined ORs in native OSNs in vivo together with high throughput assays of global OSN activity evoked by odorants at near physiological conditions in live animals^2,3^ is likely to provide the most complete understanding of the pharmacological profiles and MRRs of mammalian ORs. Understanding the mode of operation of ORs in vivo is also increasingly important given that a number of GPCRs belonging to the family of chemosensory receptors, have been directly implicated in regulating health conditions in non-sensory tissues^59^.

## Methods

### Animals

Adult 4 to 8-week old C57/Bl6 mice and Sprague–Dawley rats of both sexes were used in the study. All procedures were performed in accordance with the ARRIVE guidelines and the University of Florida IACUC-approved protocol and in accordance with the National Institutes of Health guidelines. All experiments were performed in accordance with relevant guidelines and regulations.

### rAAV2/5 design and production

To engineer the rAAV5 based bicistronic expression system we used the previously described plasmid pTR UF50-BC encoding a fluorescent protein mApple and Firefly Luciferase^60^. Two separate OR-expressing cassettes were designed: Olfr73-furin-F2A-GCaMP3, and Olfr599-furin-F2A-GCAMP, each subcloned between two AAV2 ITRs. Full-length Olfr599 (a gift from Dr. Matsunami, Duke University) and Olfr73 (a gift from Dr. Touhara, Tokyo University) were cloned in-frame with a furin protease target cleavage peptide fused to a ribosome-skipping sequence 2A, immediately followed by GCaMP3 reporter. The GCaMP3-encoding sequence was amplified by PCR from a lipid-anchored version Lck-GCaMP3 (Addgene #26974, a gift from Dr. Shigetomi). The cassette contained no additional sequence tags enhancing surface targeting of the OR, ensuring that both co-expressed open-reading frame (ORF) sequences were synthesized as precise phenocopies of their wild type counterparts. Expression of the bicistronic transcript was driven by a strong ubiquitous chicken β-actin promoter containing cytomegalovirus transcription enhancer element. Standard subcloning technique and Gibson assembly (New England Biolabs) were used to assemble inserts. All sequences were independently verified by a commercial service (IDT Inc.). For packaging rAAV5, a plasmid pXYZ5, containing all necessary helper viral genes along with the AAV vector plasmid were transfected into HEK293 cells. Following 72 h post-transfection, cells were harvested and rAAV5 was purified using an iodixanol gradient method described earlier^28^.

### In vivo imaging of bioluminescence

Mice were anesthetized with a Ketamine/Xylazine mixture and 10–15 µL of rAAV-CBA-mApple-furin2A-Luciferase (titer ca. 10^11^ vg/ml), was delivered intranasally as a single infusion per nostril. Animals were used for imaging at least 7 days post-inoculation as described previously^61^. As a control for in vivo Luciferase imaging, a small volume of the vector was injected in the tibial muscle of hind leg. For in vivo imaging, animals were anesthetized with isoflurane, intraperitoneally injected with 15 mg/mL of XenoLight potassium salt of D-luciferin (PerkinElmer) dissolved in sterile divalent-free sterile PBS, and imaged between 5 and 20 min after the injection using a Xenogen IVIS imager (Perkin Elmer). The images were analyzed and saved as TIFF files using Living Image software (PerkinElmer). Following in vivo imaging the same animal was cardiac perfused with the ice-cold 4% PFA in phosphate-buffered saline. The nasal tissue was decalcified, protected in 30% sucrose and frozen in OCT medium. Cryostat coronal sections were examined for the presence of mApple fluorescence in the olfactory epithelium.

### En face confocal imaging of the olfactory epithelium

Animals were anesthetized with CO_2_, rapidly decapitated, and the entire turbinates and septum were dissected and kept on ice in a petri dish filled with freshly oxygenated with carbogen modified artificial cerebrospinal fluid (ACSF) that contained (in mM): 120 NaCl, 25 NaHCO_3_, 3 KCl, 1.25 Na_2_HPO_4_, 1 MgSO_4_, 1.8 CaCl_2_, 15 glucose, 305 mOsm (adjusted with sucrose), pH 7.4. For imaging, a small piece of the olfactory epithelium was mounted in a perfusion chamber (RC-23, Warner Instruments) with the apical surface facing down and analyzed in a Leica SP5 confocal microscope equipped with a 63 × water-immersion objective, using a preset configuration for acquisition of mApple fluorescence.

### Single cell GCaMP3 calcium imaging

Animals 4–6 weeks of age were used for experiments 7–21 days post-inoculation with rAAV2/5 encoding OR-furin2A-GCaMP3. Calcium imaging was performed as detailed previously^31^. Tissue was prepared and mounted with the apical surface facing up. The chamber was transferred to the stage of an upright microscope (Zeiss Axioskop-2F) equipped with a 40×/0.75NA long distance water-immersion objective lens. Experimental solutions were applied for a duration of 5 s directly to the field of view through a 100 µm diameter needle made of fused silica and connected to a 9-channel Teflon manifold. Solution application was controlled by electronic valves (VC-6, Warner Instruments). The calcium response is presented as an increase of GCaMP3 fluorescence originating from the knob and underlying dendrite. The tissue was illuminated using a standard eGFP filter cube BP490 nm/ 535 nm (Omega Optical, USA) and the emitted light was collected at 530 nm (BP 530/20 nm, Omega Optical, USA) by a 12-bit cooled CCD camera (ORCA R2, Hamamatsu, Japan). Both the illumination system (Lambda DG-4, Sutter Instruments, USA) and image acquisition were controlled by Imaging Workbench 6 software (INDEC BioSystems). Before processing, fluorescence intensity was corrected for the background. Each knob was assigned a region of interest (ROI) and changes in fluorescence intensity within each ROI were analyzed and expressed as the peak fractional change in fluorescent light intensity (F-Fo)/Fo where Fo is the baseline fluorescence before application of experimental solutions.

### Transient expression of mouse ORs in HEK293 cells

Heterologous expression of mouse ORs was based on the previously published method^25^. HEK293 (ATCC CRL-1537) or HEK293T (ATCC CRL-3216) cells were grown in a high glucose Dulbecco’s minimum essential medium (DMEM) supplemented with 10% FBS, penicillin and streptomycin, and 2 mM L-glutamine and maintained at 37 °C (5% CO_2_). A rhodopsin-tagged mouse olfactory receptor Olfr73 and Olfr599 (provided by Dr. Matsunami, Duke University) was co-expressed with RTP1s, a short version of the Receptor Transporting Protein 1 ensuring proper surface trafficking of the receptor (provided by Dr. Touhara, University of Tokyo). To reconstitute different G-protein coupled signaling pathways, one of the three G-proteins was co-expressed along with the OR and RTP1s, promiscuous mouse G_α_15 (Thermo Scientific Open Biosystems), heterotrimeric G_α_q11/ß1/γ13, both coupled to endogenous PLC/calcium release, and human G_α_olf (Missouri S&T cDNA Resource Center). To assay activity-dependent accumulation of cAMP generated by activation of the Gs/Golf pathway, the cells were additionally co-transfected with a mutated A2 subunit of the rat olfactory cyclic nucleotide gated channel (CNGCmut). Two mutations C460W and E583M ensured high sensitivity and selectivity of the channel to cAMP (a gift from Dr. Rich, University of South Alabama). Transfections were performed using Calfectin (SignaGen). Cells at 70–80% confluence were co-transfected with equimolar ratios of the OR plasmid, RTP1s, the plasmid encoding each subunit of the G-protein in question, and the CNGCmut-encoding plasmid. To assay potential effects of the N-terminal modification on OR function, the cells were separately transfected with untagged version of the same OR encoded by the rAAV5 plasmid pTR-CBA-OR-furin-2A-GCaMP3. After transfection, the cells were grown for at least 48 h to allow the functional reconstitution of the cAMP-dependent pathway and the CNGCmut reporter assay.

### Calcium imaging of odorant induced response in HEK293 cells

Calcium imaging was performed as described previously^25^. HEK293 cells were incubated (30 min/37 °C) in a DMEM containing 6–8 µM Fluo-4/AM (Invitrogen) or Fluo-2/AM (TefLabs) containing 0.04% Pluronic F127. Activity of cells expressing untagged ORs from the bicistronic pTR plasmid was reported by expressed GCaMP3. Odorant solutions were applied sequentially to the cells for 5 s with a 3 min interval between each application to allow recovery from desensitization resulting from previous application of odorants. In the case of prolonged 10-min stimulation, the cells were allowed to recover for at least 10 min before adding a binary mix or isoproterenol alone, activating endogenous production of cAMP. Cells growing either on a 35-mm plastic dish or on a round poly-L-lysine coated cover glass were transferred to the stage of an inverted microscope (Axiovert 200, Zeiss) equipped with a 10x/0.5NA Fluar objective. Odorants were applied to the cells as a 5-s long pulse using a computer-controlled fast perfusion system (RSC-200, BioLogic, France) ensuring precise delivery of the stimulus with a minimal time lag. The perfusion system was controlled by Clampex 9.2 or 10.2 software (Molecular Devices). In some experiments, cells were pre-incubated with a putative antagonist for 30-s before applying a 5-s pulse of agonist. Additional measurements were performed using an inverted microscope (Olympus IX-71) equipped with a 20x/0.45NA U-Plan objective lens. Both microscopes were equipped with 12-bit cooled CCD cameras (ORCA R2, Hamamatsu, Japan). A standard FITC filter set (excitation at 510 nm, emission at 530 nm, dichroic mirror 516 nm) was used for single-wavelength measurements. The illumination system (Lambda DG-4 or Lambda L10-BS with a Smart Shutter controller, Sutter Instruments) and image acquisition were controlled by Imaging Workbench 6 software (INDEC BioSystems) under master control of Clampex software (Molecular Devices) to ensure syncing of image acquisition and odorant application. Image post-processing was performed as described above.

### Using a Cre-SEAP assay to measure cAMP production activated by odorants

cAMP production was measured as previously described^62^. HEK293T cells were co-transfected with a guanine nucleotide exchange factor Ric-8b (50 ng; a gift from Dr. Malnic, Universidade de São Paulo, Brazil), Gαolf (50 ng), RTP1s (100 ng) and untagged Olfr73 (1.5 μg). Cells were also transfected with 1.5 μg of a pCRE-SEAP, where the expression of the secreted alkaline phosphatase (SEAP) is under regulation of the cAMP responsive element. A plasmid pTAL-SEAP missing the Cre element was used as a negative control. Cells were also transfected with 50 ng of a pcDNA5/TO/LacZ (Invitrogen) for DNA concentration normalization. At 24 h post-transfection, the cells were re-seeded for SEAP analysis and odorants were added at the indicated dilutions at 48 h post-transfection. Cells and supernatants were collected 20 h later and centrifuged for 5 min at 5000 *g*. The supernatants were incubated for 30 min at 65 °C and then frozen until analysis. The cell pellets were washed with PBS and then lysed with reporter lysis buffer for β-galactosidase measurement following the manufacturer’s instructions (Promega). SEAP activity was measured by mixing 100 μl of supernatant with an equal amount of BluePhos substrate (KPL). Samples were monitored for color development at 630 nm in a microwell plate reader. SEAP activity was calculated by subtracting the responses of cells transfected in parallel with pTAL-SEAP and normalizing to β-gal activity. Average SEAP activity was determined after subtracting the response of cells not expressing an OR and is reported in arbitrary units ± standard deviation.

### Reagents, odorants and solution application

IBMX and forskolin (from Tocris or Cayman) were dissolved in DMSO and stocks kept at − 20 °C. Single odorants of the highest purity were purchased from Sigma-Aldrich, Acros Chemicals, Alpha Aesar and dissolved in anhydrous DMSO as a 0.5 M stock. For application odorants were delivered as aqueous solutions at the concentration indicated, prepared in freshly oxygenated ACSF. ACSF supplemented with 0.1% DMSO, the odorant carrier, served as the control solution. Odorant stocks were kept at − 20 °C and the final aqueous solutions were prepared on the day of the experiment in a magnesium-free Ringer’s solution containing (in mM): 140 NaCl, 5 KCl, 1.8 CaCl_2_, 0 MgCl_2_, 10 HEPES, 1.25 sodium pyruvate, 10 glucose, pH 7.6, and diluted immediately before experiments to the indicated concentration.

### Statistical analysis

Analysis and graphical presentation of the data were performed with Imaging Workbench 6 (INDEC), Microsoft Excel, Clampfit 9.2 (Molecular Devices), NIH ImageJ 1.52 (http://imagej.nih.gov/ij) and assembled in CorelDraw v.18 (Corel). Statistical analysis was performed in Microsoft Excel and Graph Pad Prism 8.

## Supplementary Information

Supplementary Video 1.

Supplementary Video 2.

Supplementary Video 3.

Supplementary Video 4.

Supplementary Information 1.
