# Supplementary material for: Mixture interactions at mammalian olfactory receptors are dependent on the cellular environment
Source: Sci Rep. 2021 Apr 29;11:9278. doi: 10.1038/s41598-021-88601-0 (PMC8085013; doi:10.1038/s41598-021-88601-0)
Supplement: Supplementary file 5 — Supplementary Information 1. [file 41598_2021_88601_MOESM5_ESM.pdf]

Supplementary Figure legends

Mixture interactions at mammalian olfactory receptors are dependent on the cellular environment

Elizabeth A. Corey[1,5], Sergei Zolotukhin[2,5], Barry W. Ache[1,3,5,6], and Kirill Ukhanov[4,5,*]

Supplementary Figure S1. Olfr599 is functionally expressed in heterologous system as well as in mouse OSNs following rAAV2/5 gene delivery. (A) A response to octanoic acid (100 µM) measured in HEK293 cells (n = 57) expressing rho-tagged Olfr599. (B) Concentration-dependence of the response yielding EC_{50} = 70 ± 40 µM (n = 3). (C) A response to octanoic acid (100 µM) measured in HEK293 cells (n = 15) co-expressing untagged Olfr599 with GCaMP3 from pTR-AAV2/5 plasmid is fully functional in heterologous system. (D) The *en face* wide-field fluorescence micrograph of the mouse OE expressing Olfr599 and GCaMP3 following one month after nasal infusion of rAAV2/5 Olfr599-furin2A-GCaMP3. Bright spots are dendritic knobs of the transduced OSNs. (E) A representative recording from five Olfr599 expressing OSNs showing elevation of GCaMP3 fluorescence evoked by application of octanoic acid and octanol (both at 100 µM). A mixture of IBMX/forskolin (100/10 µM) was applied at the end as a positive control. Traces are averaged recordings superimposed with a SEM (gray shadow). Stimulation was applied as a 5-sec pulse (triangle). Each experiment was independently replicated at least 3 times.

Supplementary Figure S2. Olfr599 heterologously expressed in HEK293 cells is functionally coupled to Gα15, Gs/olf and a heterotrimeric G-protein, Gq11/ß1/γ13. (A) Co-expression of the OR with Gα15 mediates coupling to the endogenous PLC-dependent signaling as measured by the elevation of intracellular Ca^{2+} evoked by octanoic acid (100 µM, 31 cells). (B) Co-expression of the OR with Gs/olf results in the ligand-activated calcium influx through the CNGCmut channels (23 cells). (C) Co-expression of the OR with a heterotrimeric Gq11 protein results in the ligand-evoked calcium release (43 cells). Traces represent recordings of Fluo-3 fluorescence measured in individual HEK293 cells. Stimulation was applied as a 5-sec pulse (triangle). Each experiment was independently replicated at least 3 times.

Supplementary Figure S3. Antagonism in a binary mix of eugenol and putative antagonists mediated by Olfr73 heterologously co-expressed with $G_{\alpha}15$ in HEK293. (A) A response to eugenol (100 µM),

methylisoeugenol (MIEG, 1 mM) and their binary mix applied at the indicated time and measured in 78 cells. (B) Another potent agonist, vanillin (100 µM) was antagonized by MIEG (1 mM) but required a 30-sec pre-incubation (horizontal bar) to achieve complete inhibition of the response (78 cells). (C) Dimer of isoeugenol (di-IEG, 500 µM) is not an agonist but antagonizes eugenol in a binary mix (33 cells). (D) Carvone (1 mM) was used as a negative control being a non-agonist of Olfr73 incapable even after 30 sec pre-incubation (horizontal bar) of inhibiting the response to eugenol in a binary mix (78 cells). (E) Responses in all groups were normalized to the control response evoked by eugenol alone. Data represent three independent experiments. Paired t-test, **p=0.03 (eug/di-EG); **p=0.04 (eug/di-IEG); ***p=0.0003 (Van/MIEG). Stimulation was applied as a 5-sec pulse (triangle). Traces are averaged recordings from a respective number of cells superimposed with a SEM (gray shadow).

Supplementary Figure S4. Antagonism in a binary mix of eugenol and putative antagonists mediated by Olfr73 heterologously co-expressed with Gs/olf in HEK293 (A-C). Responses mediated by a cAMP-dependent pathway. Eugenol and vanillin (both at 100 µM) are antagonized by co-application of methylisoeugenol (MIEG, 1 mM). Again, pre-incubation for 30 sec with MIEG (bar) was required to fully inhibit the response to the mixture. Carvone (Carv, 1 mM) did not activate any response and had no inhibiting effect even after 30-s preincubation (horizontal bar) on the response to eugenol. (D) Response to a single ligand and to the respective binary mix were grouped in pairs. All responses were normalized to the control response elicited by eugenol (100 µM). Data represent three independent experiments. Paired t-test, ***p=0.003 (vanillin); **p=0.02 (Van/MIEG); ***p=0.0004 (Van/MIEG, preincub). Stimulation was applied as a 5-sec pulse (triangle). Traces are averaged recordings from the same group of 31 cells superimposed with a SEM (gray shadow).

Supplementary Figure S5. Untagged Olfr73 maintains the antagonism in the binary mix of eugenol and methylisoeugenol (MIEG). (A) HEK293 cells were co-transfected with the same plasmid pTR-Olfr73-furin2A-GCaMP3 used to make rAAV2/5 and Gα15. Eugenol (100 µM) applied for 5-sec (triangle) robustly activated cells as reported by GCaMP3 whereby adding MIEG (1 mM) did not evoke any response, however significantly reduced the response to the binary mix (eug/MIEG). Traces are average of 17 cells with superimposed SEM (gray shadow). (C). Responses to MIEG and a binary mix were normalized to that evoked by eugenol alone. Paired t-test, ***p=0.0001, t=14.05, df=4, n=5.

Supplementary Figure S6. The response to eugenol measured with a cAMP-dependent Cre-SEAP reporter assay in HEK293 cells co-expressing untagged Olfr73 with Gs/olf, RTP1S and Ric8B. The data (mean ± SEM) was fitted with a Hill equation yielding EC_{50} = 205 ± 27 µM (n = 3).

Supplementary movie S1. Time-lapse series shows the response of rat OSN co-expressing Olfr73 and GCaMP3. This data was used to generate Figure 2A-B. Several sustentacular cells having much larger size than the knob, display aberrant changes of the GCaMP3 fluorescent not related to the application of eugenol. GCaMP3 fluorescence was acquired at the rate of 2 frames per second.

Supplementary movie S2. Time-lapse series shows the response of mouse OSN co-expressing Olfr73 and GCaMP3. This data was used to generate Figure 2C-D. Several sustentacular cells having much larger size than the knob, display aberrant changes of the GCaMP3 fluorescent not related to the application of eugenol. GCaMP3 fluorescence was acquired at the rate of 2 frames per second.

Supplementary movie S3. Representative time-lapse series of the response measured by GCaMP3 co-expressed in HEK293 cells along with untagged Olfr73 using rAAV2/5 plasmid pTR-Olfr73-furin2A-GCaMP3. Cells were incubated for 10 min with eugenol (100 µM) activating Gs/olf-ACIII-cAMP pathway which resulted in developing inward calcium influx through the co-expressed CNGCmut channels. GCaMP3 fluorescence was acquired at the rate of 1 frame per second.

Supplementary movie S4. The time-lapse series of the response measured in same cells showed in Movie S3. Following 15 min recovery after previous stimulation, cells were incubated for 10 min with eugenol (100 µM) mixed with methylisoeugenol (MIEG, 1 mM) which resulted in progression of the cAMP-dependent inward calcium influx through the co-expressed CNGCmut channels. GCaMP3 fluorescence was acquired at the rate of 1 frame per second.

Supplementary Figures

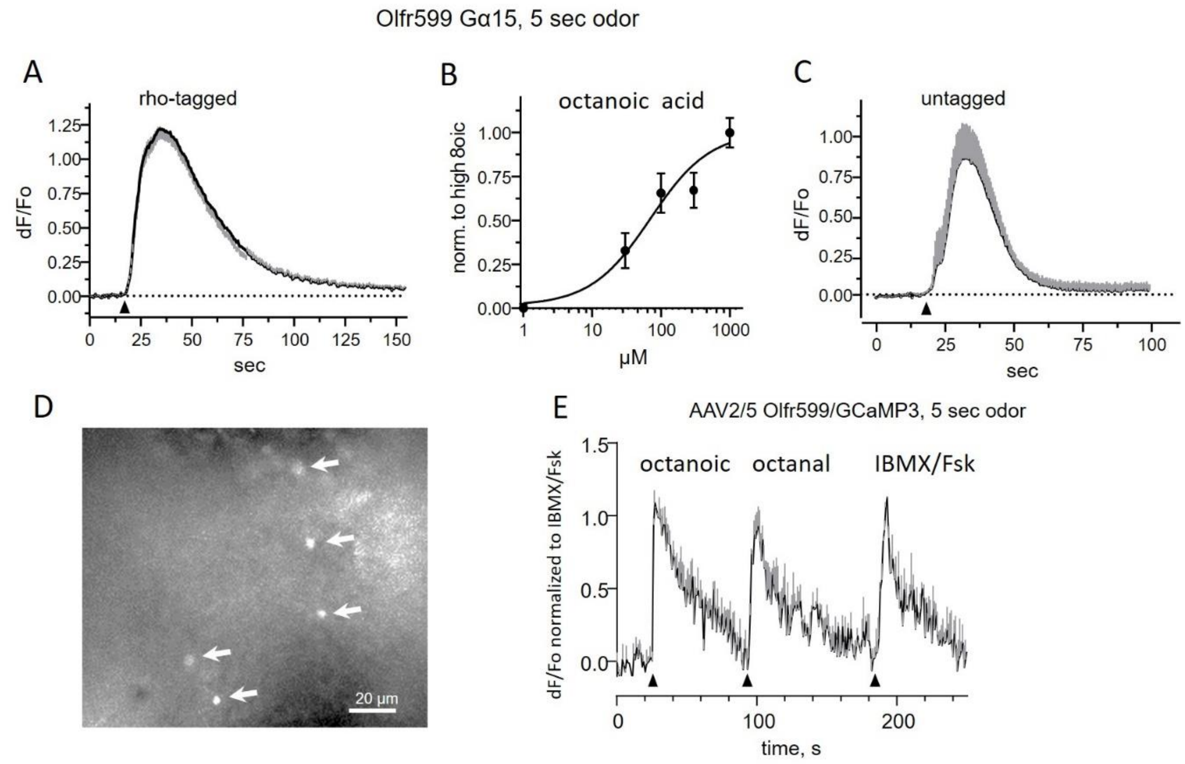

Supplementary Figure S1

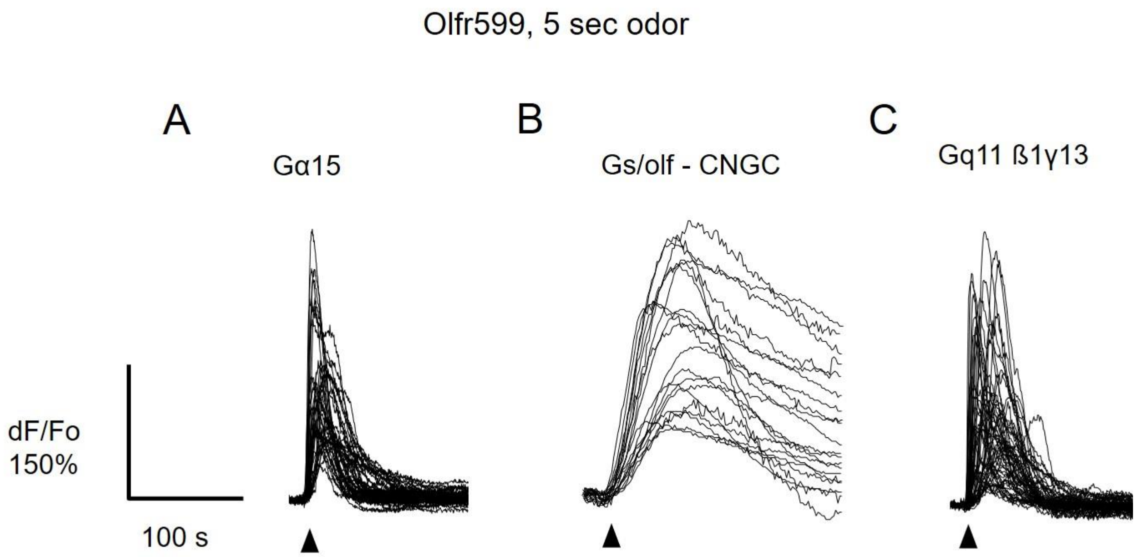

Supplementary Figure S2

Olfr73 Gα15, 5 sec odor

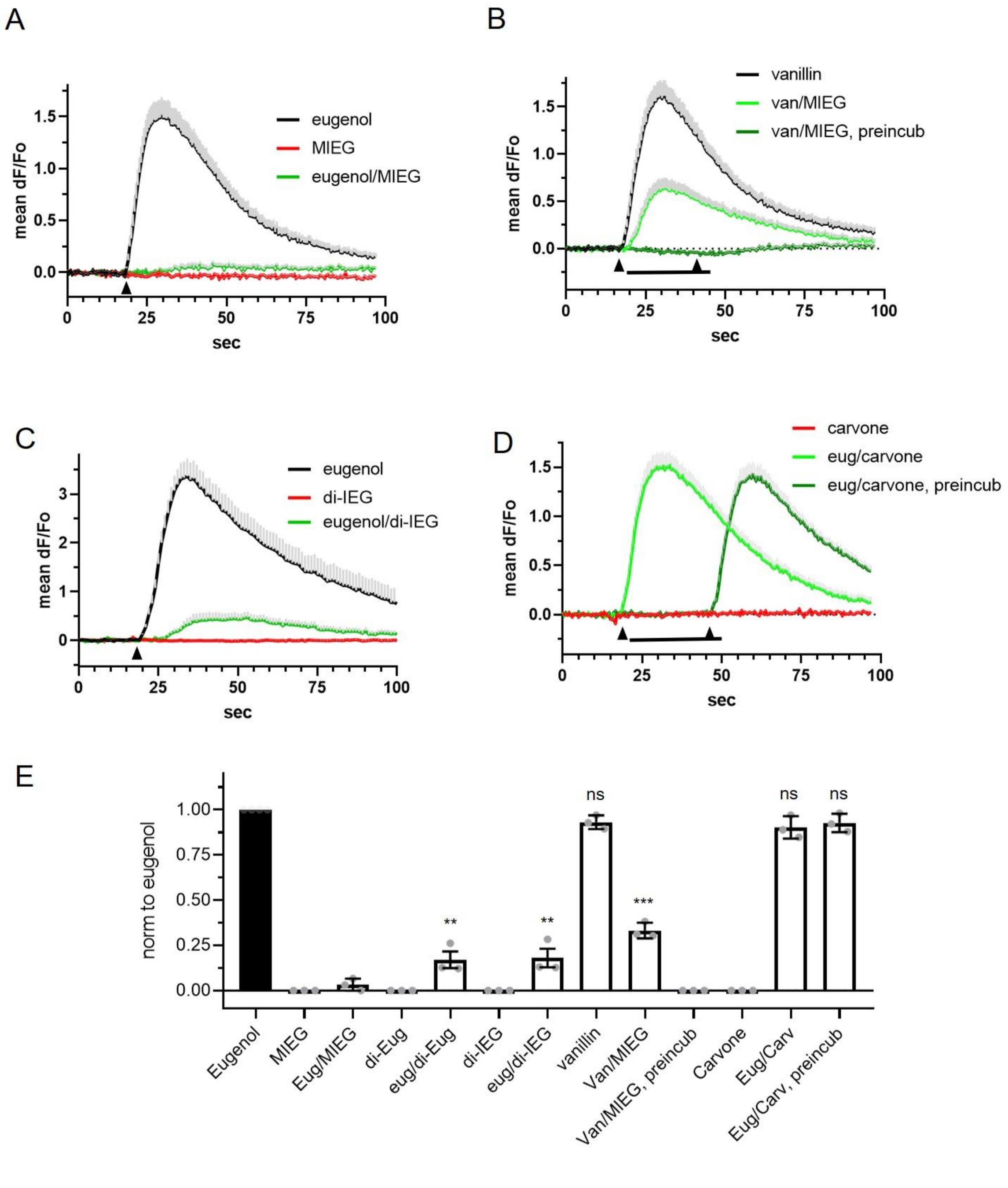

Supplementary Figure S3

Olfr73 Gs/olf; 5 sec odor

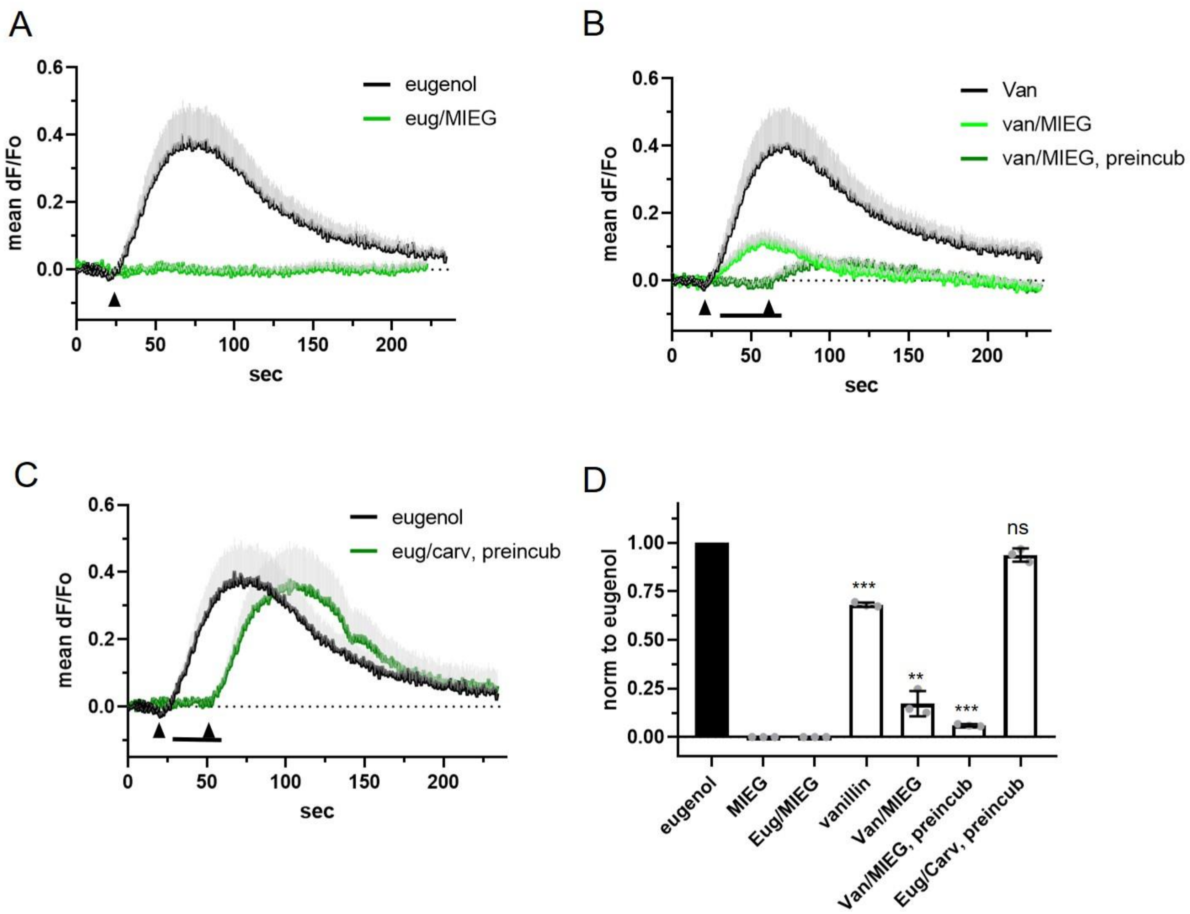

Supplementary Figure S4

pTR-Olfr73-GCaMP3 Gα15; 5 sec odor

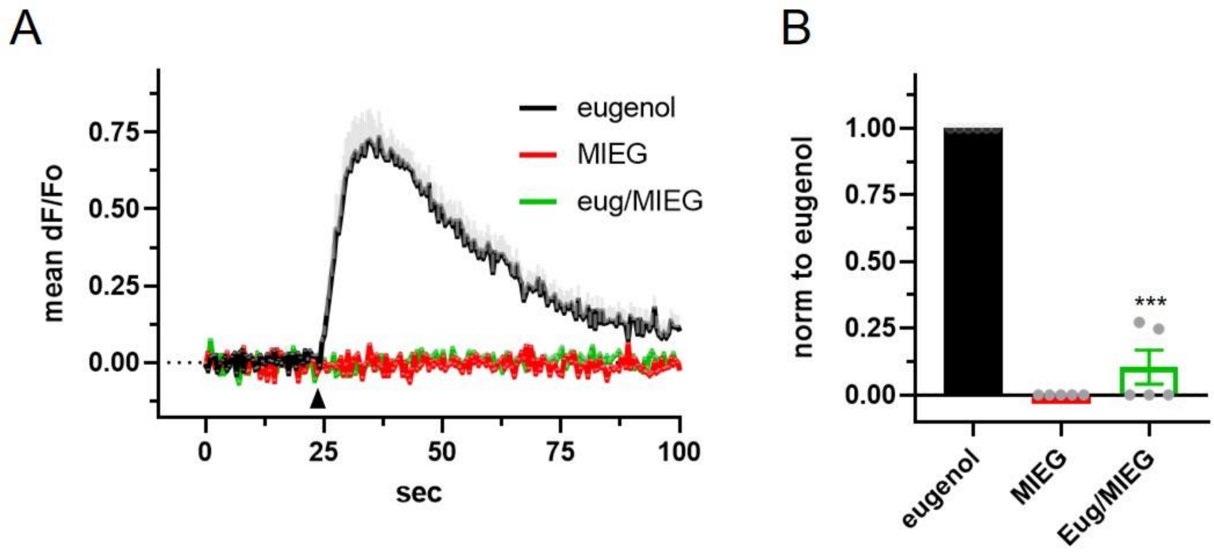

Supplementary Figure S5

untagged Olfr73 Gs/olf, 30 min odor

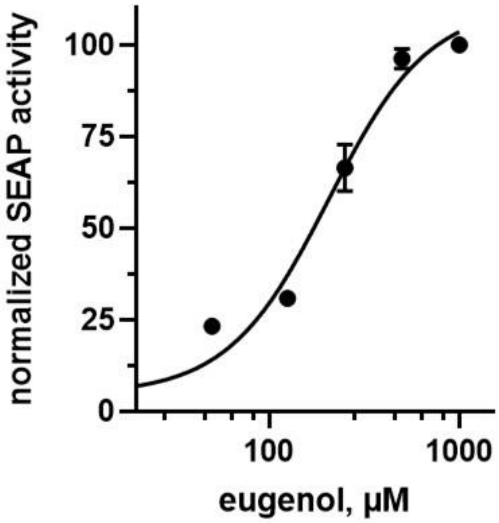

Supplementary Figure S6
